# Incidence and hemodynamic feature of risky esophageal varices with lower hepatic venous pressure gradient

**DOI:** 10.7150/ijms.37040

**Published:** 2019-11-09

**Authors:** Hitoshi Maruyama, Kazufumi Kobayashi, Soichiro Kiyono, Sadahisa Ogasawara, Yoshihiko Ooka, Eiichiro Suzuki, Tetsuhiro Chiba, Naoya Kato, Yasuyuki Komiyama, Masashi Takawa, Hiroaki Nagamatsu, Shuichiro Shiina

**Affiliations:** 1 Department of Gastroenterology, Juntendo University, 2-1-1, Hongo, Bunkyo-ku, Tokyo, 113-8421, Japan;; 2 Department of Gastroenterology, Chiba University Graduate School of Medicine, 1-8-1, Inohana, Chuo-ku, Chiba, 260-8670, Japan.

**Keywords:** esophageal varices, ultrasound, left gastric vein, portal hemodynamics

## Abstract

**Background:** To examine the incidence of cirrhosis patients with high-risk esophageal varices (EV) who show hepatic venous pressure gradient (HVPG) < 10 mmHg and to identify their hemodynamic features.

**Methods:** This prospective study consisted of 110 cirrhosis patients with EV, all with the candidate for primary or secondary prophylaxis. Sixty-one patients had red sign, and 49 patients were bleeders. All patients underwent both Doppler ultrasound and HVPG measurement.

**Results:** There were 18 patients (16.4%) with HVPG < 10 mmHg. The presence of venous-venous communication (VVC) was more frequent in patients with HVPG < 10 mmHg (10/18) than in those with HVPG ≥ 10 mmHg (19/92; p = 0.0021). The flow volume in the left gastric vein (LGV) and the incidence of red sign were higher in the former (251.9 ± 150.6 mL/min; 16/18) than in the latter (181 ± 100.5 mL/min, p = 0.02; 45/92; p = 0.0018). The patients with red sign had lower HVPG (13.3 ± 4.5) but advanced LGV hemodynamics (velocity 13.2 ± 3.8 cm/s; flow volume 217.5 ± 126.6 mL/min), whereas those without red sign had higher HVPG (16.2 ± 4.6, p = 0.001) but poorer LGV hemodynamics (10.9 ± 2.3, p = 0.002; 160.1 ± 83.1, p = 0.02).

**Conclusion:** Patients with high-risk EV with HVPG < 10 mmHg showed 16.4% incidence. Although low HVPG may be underestimated by the presence of VVC, the increased LGV hemodynamics compensates for the severity of portal hypertension, which may contribute to the development of red sign.

## Introduction

Portal hypertension is a major underlying pathogenesis of cirrhosis. It originates from increased portal inflow and/or increased outflow resistance, and the development of intra-/extrahepatic collateral vessels also affects the hemodynamic condition 1. Consequently, cirrhosis patients suffer from various complications such as gastroesophageal and ectopic varices, hepatic encephalopathy, and ascites 2.

Esophageal varices (EV) is a major complication of cirrhosis. Its frequency is approximately 30% to 40% in compensated cirrhosis and 60% in patients with ascites 3. It is also reported that the overall bleeding rate is about 25% over 2 years, and the mortality rate related to EV bleeding is about 20% 4, 5. Better understanding of the pathophysiology of risky varices may be a pivotal issue for proper management against an unfavorable event 6.

The hepatic venous pressure gradient (HVPG) is a typical surrogate marker for portal pressure; significantly increased risk of complications caused by portal hypertension is associated with HVPG >10 mmHg, defined as clinically significant portal hypertension (CSPH), and severer conditions such as variceal bleeding are linked to HVPG with 12 mmHg or more 7, 8. Moreover, the HVPG may be one of the most important parameters for patients with cirrhosis for possible prediction of their prognosis 9, 10.

However, as the HVPG value is affected by the presence of intrahepatic venous-venous communications (VVC) with the incidence from 13% to 35% in cirrhosis 11-13, it could be determined as a marker, which is subject to hemodynamic modification. Because there is such a variety of portal hemodynamics and because they are so complicated, the HVPG may not always reflect the substantial severity of portal hypertension.

Against the background, we have designed this prospective study to examine the incidence and characteristics of cirrhosis patients with high-risk EV showing HVPG < 10 mmHg, which represents a sub-CSPH condition. Further, we identified the hemodynamic features in such cases, particularly focused on the evaluation of left gastric vein (LGV), which is the main inflow route to the EV with respect to the development of red sign.

## Method

### Study

This is a newly designed cross-sectional study performed at our university hospital between December 2011 and September 2018. The study was approved by the ethical committee of our department as having an appropriate design for publication. The inclusion criteria of the study were as follows: (1) those diagnosed with cirrhosis by a laboratory test combined with two different imaging modalities (ultrasound [US] and computed tomography [CT]/magnetic resonance imaging [MRI]); (2) those who were candidates for primary prophylaxis, with medium- or large-grade EV, and/or any-grade EV with red sign diagnosed by endoscopy; (3) those who were candidates for secondary prophylaxis; (4) those with no history of EV treatment; (5) those with no history of beta-blocker medication (it is not approved for portal hypertension in our country); and (6) those who were scheduled for hepatic venous catheterization. If the patient decided to participate in the study, Doppler US for the assessment of portal hemodynamics was performed at the time of admission for hepatic venous catheterization, and upper gastrointestinal endoscopy was conducted following Doppler examination.

However, the study excluded the following patients: (1) those with advanced hepatocellular carcinoma (HCC) showing C/D stage by the Barcelona-Clinic Liver Cancer Staging System 14; (2) those with portal vein obstruction caused by thrombus or tumor thrombus, cavernoma, or intrahepatic arterioportal shunt detected by US and/or CT/MRI; (3) When hepatic venography showed the findings characteristic to idiopathic portal hypertension typified by weeping willow appearance or no retrograde detection of intrahepatic portal vein 15, the patient was not included in the study because of the suspicion of idiopathic portal hypertension.; (4) those with a history of abdominal surgery, partial splenic embolization, or transjugular intrahepatic portosystemic shunt; and (5) those who were pregnant at the time of the study.

### Ultrasound

The study used an SSA-770A or 790A (Toshiba, Tokyo, Japan) with a 3.75-MHz convex probe. The operator (H.M.) had more than 20 years of US experience and was blinded to the endoscopic findings. The patients underwent US examination in the supine position after fasting for 4 hours or more. After the routine observation, the portal system was carefully assessed, and the diameter and the velocity in the portal trunk and in the main part of the LGV, and in the other collaterals were measured. Briefly, the pulsed Doppler technique used the sampling width corresponding to the vessel diameter, and the blood flow was assessed at an angle < 60 degrees between the US beam and the vessel. The mean flow volume (mL/min) was calculated by determining the mean velocity for 1 second for the cross-section of the vessel and multiplying it by 60 seconds 16.

The spleen size (mm^2^) was determined by multiplying the distance from the splenic hilum to the caudal polar angle, measured with two intersecting lines, according to the literature 16. The upper limit of normal used in the study was 2000 mm^2^. The data used for analysis were the average values, which were calculated using measurements taken 2 to 4 times.

The presence or absence or the degree of ascites was assessed based on clinical and US findings. Mild ascites was defined as that detectable only by US examination, moderate ascites was defined as that causing moderate symmetrical distention of the abdomen, and severe ascites was defined as that causing marked abdominal distension.

### Endoscopy

Endoscopic examination was performed using a GIF-H260 or GIF-Q240 system (Olympus Corp., Tokyo, Japan) and was performed by either S.K. or K.K, each of whom had more than 7 years of experience and were blinded to the US findings. Gastroesophageal varices were classified as small, medium, or large 17. In addition, the presence or absence of red sign and portal hypertensive gastropathy (PHG) were assessed 17.

The study defined EV bleeding by the presence of both of the following findings: (i) an apparent bleeding history and (ii) endoscopic evidence of active bleeding or a fibrin clot on the varices. However, even in cases without evidence of active bleeding or a fibrin clot, the varices were considered to be the source of bleeding when no other cause for gastrointestinal bleeding could be identified.

### Hepatic Venous Catheterization

The study performed hepatic venous catheterization with the standard method 18. Free and wedged hepatic venous pressure were measured in the right main hepatic vein using a balloon catheter (5 Fr, 9 mm; Terumo Clinical Supply Co. Ltd, Gifu, Japan), and the HVPG was calculated as the difference between them. The presence or absence of VVC was assessed by two independent reviewers, H.M and S.K. or K.K. When VVC was detected on the venogram, the measurement was repeated in a different position of the hepatic vein and in a different hepatic vein.

### Statistical Analysis

Student's t test, analysis of variance, or Pearson product-moment correlation coefficient was used for continuous variables, and Fisher's exact test or chi-square test was used for categorical variables. A p-value <0.05 was considered statistically significant. The statistical values were calculated using SAS software (SAS Institute Inc., Cary, NC, USA).

## Results

### Patient Characteristics

The study included 110 cirrhosis patients (73 male, 37 female) with an average age of 62.4 years (standard deviation [SD], 12.0; range, 23-86; Table 1). The degree of EV by endoscopy was small in 13 patients, medium in 58 patients, and large in 39 patients. Sixty-one patients (55.5%) had red sign, and 49 patients (44.5%) were bleeders. Seventy-three patients had cardiac varices, and 16 had gastric fundal varices. Liver function reserve presented by Child-Pugh classification was A in 61, B in 41, and C in 8. The HVPG ranged from 2.9 to 30.3 mmHg (14.6 ± 4.8).

The diameter (mm), velocity (cm/s), and flow volume (mL/min) in the portal trunk and in the LGV are summarized in Table 2. The median interval between US examination and hepatic venous catheterization was 1 day (range, 0-3 days), between bleeding episode and US examination was 11 days (range, 0-20 days), and between bleeding episode and hepatic venous catheterization was 15 days (range, 3-29 days).

### HVPG and Clinical Findings

There were 18 patients (16.4%) with HVPG < 10 mmHg (2.9-9.9, median 8.6) and 92 patients (83.6%) with HVPG ≥ 10 mmHg (10.3-30.3, median 15.1) (Table 3). Liver function reserve showed no difference between the two groups.

**Figure 1:**
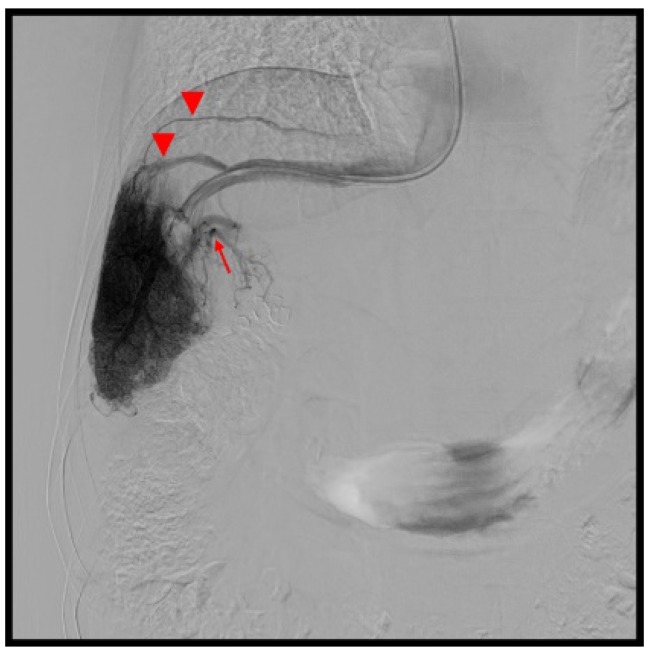
53-year old male, hepatitis C related cirrhosis. Hepatic venography showed hepatic veins (arrow heads) demonstrated via venous-venous communications. The hepatic venous pressure gradient was 9.6 mmHg. (Arrow, intrahepatic portal vein)

The presence of VVC was more frequent in patients with HVPG < 10 mmHg (10/18, 55.6 %) than in those with HGPV ≥ 10 mmHg (19/92, 20.7%; p = 0.0021) (Figure 1). The HVPG was lower in patients with VVC (n = 29; 12.6 ± 5.6 mmHg, 2.9-24.3) than in those without VVC (n = 81; 15.3 ± 4.3 mmHg, 7.4-30.3; p = 0.009). However, there was no difference in the incidence of extrahepatic portosystemic shunt between the two HVPG-related groups (5/18 vs. 30/92; p = 0.69).

As for the variceal findings, there were no differences in the degree of EV, bleeding rate, incidence of cardiac/fundal varices, and PHG between the two groups. However, red sign was more frequent in patients with HVPG < 10 mmHg (16/18, 88.9%) than in those with HVPG ≥ 10 mmHg (45/92, 48.9%; p = 0.0018). There was no significant difference in the bleeding rate in the cohort with red sign between patients with HVPG < 10 mmHg (6/16, 37.5%) and those with HVPG ≥ 10 mmHg (24/45, 53.3%; p = 0.28).

### Relationship between HVPG and LGV Parameters With Respect to Clinical Findings

**Figure 2:**
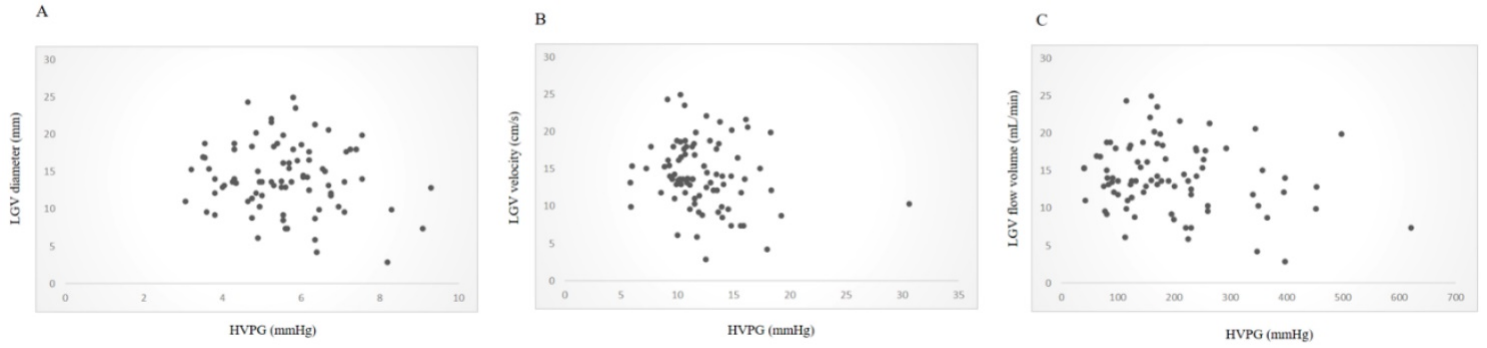
Correlation between HVPG and LGV parameters. The diameter and velocity in the LGV showed no correlation with HVPG. However, the flow volume in the LGV and the HVPG showed mild negative correlation (r = -0.21, p = 0.05). A, Diameter and HVPG (r = -0.13, p = 0.24). B, Velocity and HVPG (r = -0.18, p = 0.98). C, Flow volume and HVPG (r = -0.21, p=0.05). HVPG, hepatic venous pressure gradient; LGV, left gastric vein.

The LGV was successfully detected in 95 patients (95/110, 86.4%); 85 with reverse flow direction, 5 with bidirectional flow, and 5 with forward flow direction. Therefore, the LGV hemodynamics was assessed in the 85 patients with LGV showing reverse flow direction (52/85 with positive red sign on EV). The LGV flow volume showed difference between patients with HVPG < 10 mmHg (251.9 ± 150.6 mL/min) and those with HVPG ≥10 mmHg (181 ± 100.5 mL/min; p = 0.02), although the diameter and the velocity showed no difference (Table 3). The HVPG and the flow volume in the LGV showed mild negative correlation (r = -0.21) with marginal difference (p = 0.05; Figure 2).

There was a significant difference in the degree of EV (p = 0.0003) between patients with and without red sign (Table 4). The patients with red sign had lower HVPG (13.3 ± 4.5 mmHg) but advanced LGV hemodynamics (velocity 13.2 ± 3.8 cm/s; flow volume 217.5 ± 126.6 mL/min), whereas those without red sign had higher HVPG (16.2 ± 4.6 mmHg, p = 0.001) but poorer LGV hemodynamics (10.9 ± 2.3, p = 0.002; 160.1 ± 83.1, p = 0.02), although the LGV diameter showed no difference (5.4 ± 1.3 mm vs. 5.7 ± 1.3 mm; p = 0.38). There were no differences in the clinical background, variceal findings, or portal parameters between bleeders and nonbleeders (Table 5).

## Discussion

Doubtlessly, the HVPG is a popular and representative marker for the severity of portal hypertension. However, as shown in the previous reports, there is a certain incidence of patients with EV showing lower HVPG 19-21. The present study may be the first to focus on the clinical features of high-risk EV with sub-CSPH condition, which showed 16.4% incidence with median HVPG value of 8.6 mmHg. Because of the high detectability of VVC (55.6%), although it is unavoidable with hepatic venous catheterization, the HVPG may be underestimated in these patients.

Our study demonstrated a unique hemodynamic feature in patients with high-risk sub-CSPH EV, which is an increased flow volume in the LGV, which may compensate for the severity of portal hypertension against lower HVPG. The close linkage between the LGV hemodynamics and the development of red sign has not been discussed elsewhere and may support the presence of risky varices even under the lower HVPG condition. A significant difference in the flow volume between the two HVPG-related groups may suggest the substantial hemodynamic effect of the LGV because it reflects both the diameter and velocity according to the calculation formula. Needless to say, the fundamental research base of the present work is the scientific evidence of more than 80% detectability of the LGV by US in patients with EV 22. The present study may enhance the practical application of Doppler sonography as a noninvasive tool to predict patients with high-risk EV accompanied with red sign.

The incidence of red sign is reported to be 45.4% with small varices, 65.0% with mid-size varices, and significant factors showing a close correlation with red sign are the number of varices, size of varices, platelet count, and alpha-fetoprotein level 23. However, because no study had been performed regarding the influence of hemodynamics on the red sign, the present study first demonstrates the substantial effects of velocity and flow volume in the LGV on the development of red sign.

A red sign is considered to be related to wall thickness, which determines the wall tension of varices 1. Vascular response to the shared stress caused by a change in velocity is a well-known physiological phenomenon 24, 25, and increased flow velocity results in endothelial damage in the arterial system 26. However, there may be some common pathophysiologic conditions in the arterial and the portal venous system that result in the formation of red sign. In addition, the present study presented a trade-off like relationship between HVPG and LGV hemodynamics with respect to the presence of red sign, which may suggest the importance of local but not systemic factors for the formation of red sign.

Variceal bleeding occurs when the tension exerted by the variceal wall exceeds the rupture point 1, and it is unlikely to occur unless the HVPG exceeds 12 mmHg 27, 28. However, there is an argument about the relationship between HVPG and variceal bleeding because HVPG did not differ between patients with previous bleeds and those without bleeds with HCV-related or alcoholic cirrhosis reported by Bellis et al 29. The present study also showed no significant difference in the HVPG between bleeders and nonbleeders. However, the data may not deny the role of HVPG because all subjects in our study were candidates for prophylactic treatment, which is, having a potentially severe condition of portal hypertension. A bleeding and/or red sign of EV may occur with additional factors, even in patients with HVPG less than 10 mmHg; advanced hemodynamics in the LGV may be one of the factors.

The major limitation of our study is that there is a potential bias regarding the patient cohort. That is, the study required HVPG data as an important parameter for severity of portal hypertension. Because hepatic venous catheterization is the standard procedure before variceal treatment in our institution, the study did include patients with advanced varices alone. Further studies may be necessary to elucidate our data in a larger cohort, including patients with small EV without red sign who are not candidates for prophylactic treatment. Another limitation is that the study did not examine the relationship between the liver stiffness and the EV with lower HVPG, which needs to be clarified sometime soon.

In conclusion, there was 16.4% incidence of high-risk EV with sub-CSPH condition. Although low HVPG may be underestimated by the presence of VVC, the increased LGV hemodynamics compensate for the severity of portal hypertension, which may contribute to the development of red sign. The actual incidence and natural history of such patients in the cirrhosis cohort need to be elucidated in the future.

## Figures and Tables

**Table 1:** Patient characteristics

Number	110
Age	62.4 ± 12.0 (23-86)
Sex (Male/Female)	73 / 37
Body mass index (kg/m^2^)	23.4 ± 4.0 (14.7-35.5)
Etiology, HCV/HBV/HBV+HCV/alcohol/NASH/PBC/ PSC/NBNC	31 / 8 / 2 / 23 / 18 / 13 / 3 / 12
**Esophageal varices, n (%)**	
Small	13 (11.8%)
Medium	58 (52.7%)
Large	39 (35.5%)
Red sign, -/+	49 / 61
Bleeding, -/+	61 / 49
Cardiac varices, -/+	37 / 73
Gastric fundal varices, -/+	94 / 16
Portal hypertensive gastropathy, -/+	71 / 39
Ascites, -/mild/moderate to severe	68 / 30 / 12
Splenomegaly, -/+	17 / 93
Hepatocellular carcinoma, -/+	82 / 28
**Blood test**	
Platelet count (×10^9^/L)	84.9 ± 55.8 (19-391)
Aspartate aminotransferase (IU/L)	50.4 ± 42.5 (3-420)
Alanine aminotransferase (IU/L)	35.3 ± 47.1 (2-501)
Albumin (g/dL)	3.4 ± 0.6 (1.8-4.8)
Total bilirubin (mg/dL)	1.6 ± 1.0 (0.4-8.2)
Prothrombin time (%)	80 ± 15.8 (38-128)
Child-Pugh score	6.8 ± 1.6 (5-13)
Child-Pugh classification A/B/C	61 / 41 / 8
Hepatic venous pressure gradient	14.6 ± 4.8 (2.9-30.3)

Data are expressed as number or mean ± standard deviation (range). *HCV* hepatitis C virus, *HBV* hepatitis B virus, *NASH* non-alcoholic steatohepatitis, *PBC* primary biliary cholangitis, *PSC* primary sclerosing cholangitis, *NBNC*, non B non C.

**Table 2:** Measurement data in the portal trunk and in the left gastric vein

	Diameter (mm)	Velocity (cm/s)	Flow volume (mL/min)
Portal vein (Forward flow, n=107*)	11.4 ± 1.6 (8.1 - 15.8)	12.1 ± 2.4 (6.3 - 19.4)	763.1 ± 277.8 (237.5 - 1825)
Left gastric vein (Reverse flow, n=85**)	5.5 ± 1.4 (2.7 - 9.3)	12.1 ± 3.5 (5.8 - 30.6)	193 ± 118 (40 - 620.5)

Data are expressed as number or mean ± standard deviation (range). *, Three patients with portal trunk showing bidirectional flow direction were excluded. **, Twenty-five patients were excluded (15 with no detection of left gastric vein, 5 with bidirectional flow direction, 5 with forward flow direction).

**Table 3:** Comparison of clinical findings with respect to HVPG

	Hepatic venous pressure gradient		P value
	< 10 mmHg (2.9-9.9, median 8.6)	10 mmHg ≤ (10.3-30.3, median 15.1)	
Number	18 (16.4%)	92 (83.6%)	
Age	62.4 ± 12.5 (39-78)	62.4 ± 12.0 (23-86)	0.99
Sex (Male/Female)	12 / 6	61 / 31	0.98
Body mass index (kg/m^2^)	22.9 ± 4.3 (14.8-31)	23.5 ± 3.9 (14.7-35.5)	0.61
Etiology, HCV/HBV/HBV+HCV/alcohol/NASH/PBC/ PSC/NBNC	5 / 1 / 0 / 3 / 5 / 3 / 1 / 0	26 / 7 / 2 / 20 / 13 / 10 / 2 / 12	0.57
Esophageal varices, n (%)			0.78
Small	2 (11.2%)	11 (12.0%)	-
Medium	8 (44.4%)	50 (54.3%)	-
Large	8 (44.4%)	31 (33.7%)	-
Red sign, -/+	2 / 16	47 / 45	0.0018
Bleeding, -/+	11 / 7	50 / 42	0.6
Cardiac varices, -/+	4 / 14	33 / 59	0.26
Gastric fundal varices, -/+	16 / 2	78 / 14	0.65
Portal hypertensive gastropathy, -/+	11 / 7	60 / 32	0.74
Ascites, -/mild/moderate to severe	11 / 6 / 1	57 / 24 / 11	0.6
Splenomegaly, -/+	2 / 16	15 / 77	0.58
Hepatocellular carcinoma, -/+	15 / 3	67 / 25	0.35
Portal vein thrombosis, -/+	18 / 0	88 / 4	0.37
VVC, -/+	8 / 10	73 / 19	0.0021
Left gastric vein	(n=16)	(n=69)	
Diameter	6.1 ± 1.5 (3.6-9.1)	5.5 ± 1.2 (3.05-9.3)	0.08
Velocity	13.4 ± 3.2 (5.85-19.25)	12.0 ± 3.5 (5.8-30.6)	0.13
Flow volume	251.9± 150.6 (77.5-620.5)	181 ± 100.5 (40-497.5)	0.02
Extrahepatic portosystemic shunt, -/+	13 / 5*	62 / 30**	0.69
Blood test			
Platelet count (×10^9^/L)	63.7 ± 33.1 (29-176)	89.1 ± 58.4 (19-391)	0.08
Aspartate aminotransferase (IU/L)	43.6 ± 14.5 (27-87)	51.7 ± 46 (3-420)	0.46
Alanine aminotransferase (IU/L)	29.2 ± 11.6 (2-56)	36.4 ± 51.2 (9-501)	0.55
Albumin (g/dL)	3.6 ± 0.5 (2.6-4.5)	3.3 ± 0.5 (1.8-4.8)	0.06
Total bilirubin (mg/dL)	1.5 ± 0.6 (0.9-2.7)	1.6 ± 1.1 (0.4-8.2)	0.7
Prothrombin time (%)	81.5 ± 11.9 (64-106)	79.7 ± 16.5 (38-128)	0.66
Child-Pugh score	6.3 ± 1.1 (5-9)	6.8 ± 1.7 (5-13)	0.2
Child-Pugh classification A/B/C	11 / 7 / 0	50 / 34 / 8	0.43

Data are expressed as number or mean ± standard deviation (range). HCV hepatitis C virus, HBV hepatitis B virus, NASH non-alcoholic steatohepatitis, PBC primary biliary cholangitis, PSC primary sclerosing cholangitis, NBNC, non B non C, VVC venous-venous communications (assessment by hepatic venogram). *, splenorenal shunt 4 and short gastric vein 1. **, splenorenal shunt 19, short gastric vein 10 (1 with splenorenal shunt), and inferior mesenteric vein 4 (1 with splenorenal shunt, 1 with short gastric vein).

**Table 4:** Comparison of clinical findings between patients with and without red sign

	Red sign		P value
	- N=49	+ N=61	
Esophageal varices Small/Medium/Large	10/31/8	3/27/31	0.0003
Bleeding, -/+	30 / 19	31 / 30	0.28
VVC, -/+	39 / 10	42 / 19	0.2
HVPG (mmHg)	16.2 ± 4.6 (3.7-30.3)	13.3 ± 4.5 (2.9-22.4)	0.001
Left gastric vein	(n=33)	(n=52)	
Diameter	5.4 ± 1.3 (3.05-9.3)	5.7 ± 1.3 (3.5-9.1)	0.38
Velocity	10.9 ± 2.3 (5.85-16.1)	13.2 ± 3.8 (5.8-30.6)	0.002
Flow volume	160.1± 83.1 (40-453)	217.5 ± 126.6 (40-620.5)	0.02
Child-Pugh score	6.9 ± 1.9 (5-13)	6.6 ± 1.4 (5-12)	0.4
Child-Pugh classification A/B/C	27 / 16 / 6	34 / 25 / 2	0.17

Data are expressed as number or mean ± standard deviation (range). VVC, venous-venous communications (assessment by hepatic venogram). HVPG, hepatic venous pressure gradient.

**Table 5:** Comparison of clinical findings between bleeders and non-bleeders

	Bleeding		P value
	- (n=61)	+ (n=49)	
Esophageal varices Small/Medium/Large	7/30/24	6/28/15	0.63
Red sign, -/+	30 / 31	19 / 30	0.28
VVC, -/+	43 / 18	38 / 11	0.4
HVPG (mmHg)	14.7 ± 5.1 (2.9-30.3)	14.4 ± 4.4 (3.7-23.5)	0.7
Left gastric vein	(n=51)	(n=34)	
Diameter	5.7 ± 1.3 (3.5-9.3)	5.5 ± 1.4 (3.05-9.1)	0.55
Velocity	12.0 ± 3.9 (5.8-30.6)	12.7 ± 2.6 (7.65-18.35)	0.4
Flow volume	192.8± 108 (40-497.5)	197.6 ± 124.9 (40-620.5)	0.85
Child-Pugh score	6.6 ± 1.7 (5-13)	6.9 ± 1.5 (5-11)	0.3
Child-Pugh classification A/B/C	37 / 19 / 5	24 / 22 / 3	0.33

Data are expressed as number or mean ± standard deviation (range). VVC, venous-venous communications (assessment by hepatic venogram). HVPG, hepatic venous pressure gradient.

## Abbreviations

EV: esophageal varices
HVPG: hepatic venous pressure gradient
CSPH: clinically significant portal hypertension
VVC: venous-venous communications
LGV: left gastric vein
US: ultrasound
CT: computed tomography
MRI: magnetic resonance imaging
HCC: hepatocellular carcinoma
PHG: portal hypertensive gastropathy
SD: standard deviation
